# Anxiety disorders in the Middle East and North Africa region; 1990 to 2021

**DOI:** 10.1017/neu.2025.10023

**Published:** 2025-06-30

**Authors:** Reza Aletaha, Ali-Asghar Kolahi, Zahra Mousavi, Mark J.M. Sullman, Saeid Safiri

**Affiliations:** 1 Social Determinants of Health Research Center, Department of Community Medicine, Faculty of Medicine, Tabriz University of Medical Sciences, Tabriz, Iran; 2 Social Determinants of Health Research Center, Shahid Beheshti University of Medical Sciences, Tehran, Iran; 3 Department of Psychiatry, Faculty of Medicine, Tabriz University of Medical Sciences, Tabriz, Iran; 4 Department of Life and Health Sciences, University of Nicosia, Nicosia, Cyprus; 5 Department of Social Sciences, University of Nicosia, Nicosia, Cyprus; 6 Neurosciences Research Center, Aging Research Institute, Tabriz University of Medical Sciences, Tabriz, Iran; 7 Department of Genetics and Bioengineering, Yeditepe University, Istanbul, Turkey

**Keywords:** Anxiety Disorders, mental health, epidemiology, disability, risk factors

## Abstract

**Objective:**

This study presents the most recent data on the incidence, prevalence, and years lived with disability (YLDs) due to anxiety disorders across the Middle East and North Africa (MENA) region from 1990–2021, analysed by sex, age, and sociodemographic index (SDI).

**Methods:**

We reported the burden of anxiety disorders using data sourced from the Global Burden of Disease 2021 study. The estimates of prevalence, DALYs, and YLDs are provided as numbers and age-standardised rates, accompanied by their 95% uncertainty intervals (UIs).

**Results:**

In 2021, the age-standardised point prevalence of anxiety disorders in the region was 5.95 thousand, with an incidence rate of 883.4 per 100,000. The number of YLDs in 2021 reached 4.5 million. From 1990 to 2021, the burden of anxiety disorders increased significantly. Lebanon had the highest burden in 2021. Among both sexes, the 10–14 age group had the highest incidence rate, while the 15–19 age group had the highest prevalence and YLD rates. In 2021, most age groups in the MENA region had YLD rates that were higher than the global average.

**Conclusion:**

This study highlights the urgent need for a multidisciplinary approach to prevent and manage anxiety disorders. Ensuring accessible and affordable treatment options for all affected individuals is crucial. Governments should prioritise supporting programmes to effectively address mental health issues, given the unique socioeconomic and geopolitical challenges in the MENA region. By including effective preventive methods alongside treatment in healthcare strategies, the burden of anxiety disorders can be significantly reduced.

Significant outcomes

- The burden of anxiety disorders has increased significantly in the MENA region between 1990 and 2021. This highlights a growing public health concern that requires urgent attention.
- There are significant variations in anxiety disorder rates among MENA countries, with Lebanon, Tunisia, and Iran experiencing disproportionately higher burdens compared to others. Socioeconomic factors and specific stressors in these countries likely play a significant role.
- Women experience a higher prevalence and burden of anxiety disorders than men, potentially exacerbated by socioeconomic factors, gender socialisation, and increased caregiving responsibilities, particularly during crises like the COVID-19 pandemic.

Limitations

- The study relies on data from high-income countries to model the impact of COVID-19 on anxiety disorders in the MENA region, and the lack of robust raw data in many locations is a significant limitation.
- The aggregation of all anxiety disorders into a single category limits the understanding of the specific burdens associated with individual disorders. This aggregation might bias public health policies and resource allocation.
- The quality of data from conflict-affected regions (Syria, Yemen, Lebanon) may be compromised due to ongoing violence, restricted access, and political instability, potentially leading to incomplete or biased information.

## Introduction

Anxiety disorders are characterised by severe and persistent feelings of fear and discomfort, which are often accompanied by physical symptoms (Penninx *et al*., 2021). These disorders often begin in childhood and follow a recurring, intermittent course (Kessler *et al*., 2009), leading to significant health loss, role impairment (Mendlowicz & Stein, 2000), and lifelong disadvantages in areas such as education, income, and interpersonal connections (Lochner *et al*., 2003). Using data from the Global Burden of Diseases (GBD) 2019 Study, research has estimated that anxiety disorders are responsible for around 28.68 million disability-adjusted life years (DALYs) worldwide, with a particularly significant impact on individuals aged 10–24 years (Vos *et al*., 2020). In the MENA region, anxiety disorders rank among the top three mental disorders in terms of DALY rates (Effatpanah et al., 2024). Furthermore, research indicates that one in 14 individuals meets the criteria for the diagnosis of an anxiety disorder (Craske & Stein, 2016). The number of anxiety disorder cases grew from 31.13 million in 1990 to 45.82 million in 2019, representing an increase of about 47%, while DALYs rose by nearly 54% (18.7 million to 28.7 million cases) over the same period (Xiong *et al*., 2022). Moreover, the global age-standardised DALY rate (ASDR) for anxiety disorders increased by 16.7% between 2010 and 2021, indicating that this trend is ongoing (2024b). This increasing trend was documented in the MENA region, as the age-standardised prevalence, incidence, and DALY rates of anxiety disorders rose by 3.74%, 4.12%, and 3.81%; respectively (Effatpanah *et al*., 2024). The COVID-19 pandemic has exacerbated this situation by amplifying factors associated with poor mental health (2021).

The economic impact of anxiety disorders on society is substantial. High costs for inpatient treatment, outpatient appointments, prescription medications, and emergency care contribute to the significant financial burden of treating these mental health conditions (Shirneshan *et al*., 2013). In the United States, the additional annual direct medical costs attributed to anxiety disorders for the ambulatory adult population were $33.71 billion in 2013 (Shirneshan *et al*., 2013). A study conducted in European countries to assess the economic impact of mental disorders in 2010 revealed that the total cost of anxiety disorders exceeded €74.4 billion when adjusted for purchasing power parity (Gustavsson *et al*., 2011).

Individuals demonstrate greater willingness to pursue evidence-based psychological and pharmacological treatments when immediate benefits or reduced losses are perceptible. While medical intervention remains preferable for incidence reduction, the implementation of effective preventive measures may prove more impactful in curtailing avoidable treatment costs (Baxter *et al*., 2014).

Given the substantial economic burden of anxiety disorders, characterised by high treatment and care-related costs, it is increasingly important to have comprehensive data on their incidence, DALYs, and trends across different countries and regions. This information is essential for fully assessing the disease burden, enabling decision-makers to allocate limited resources effectively and develop informed policies based on robust evidence (Xiong *et al*., 2022). Moreover, the intricacy of mental illness, shaped by factors including socioeconomic status, political instability, conflict, violence, immigration, discrimination, and restricted access to mental health services, highlights the distinct challenges encountered by individuals, communities, and generations in the MENA region (Nagi *et al*. 2025).

Despite the recognition of anxiety disorders as a major public health issue, few studies have quantified the burden at the global, regional, national, or sociodemographic (SDI) levels. The Global Burden of Disease (GBD) study 2021, coordinated by the Institute for Health Metrics and Evaluation (IHME), offers comprehensive data on the burden of diseases, injuries, and risk factors across 204 countries from 1990 to 2021 (2024b). However, there have been no recent studies on the burden of anxiety disorders in the Middle East and North Africa (MENA) region. This study aims to fill this gap by providing the most recent information on the incidence, prevalence, and DALYs associated with anxiety disorders in the 21 nations that comprise the region from 1990 to 2021, analysed by gender, age, aetiology, and SDI.

## Methods

### Overview

GBD 2021 represents the most recent iteration of the GBD project, which is overseen by IHME (2024b). For a comprehensive understanding of the methodologies employed in GBD 2021, as well as the modifications introduced since the 2019 iteration, readers are encouraged to consult additional resources (2024b). Those interested in exploring estimates related to both fatal and non-fatal injuries can access detailed data through the following online platforms: https://vizhub.healthdata.org/gbd-compare/ and https://ghdx.healthdata.org/gbd-results-tool.

### Case definition and data inputs

All instances of anxiety disorders that met the diagnostic criteria established by the International Classification of Diseases (ICD-10), World Health Organization (WHO), or the Diagnostic and Statistical Manual of Mental Disorders (DSM-IV-TR) were included (2024b). The respective identification codes are as follows: ICD-10: F40-42, F43.0, F43.1, F93.0-93.2, F93.8; DSM-IV-TR: 300.0-300.3, 208.3, 309.21, 309.81. Post-traumatic stress disorder (PTSD), overanxious disorder in childhood, panic disorder, specific phobia, obsessive–compulsive disorder (OCD), agoraphobia, generalised anxiety disorder (GAD), social phobia, including separation anxiety disorder (SAD), and anxiety disorder ‘not otherwise specified’ (NOS) were included as anxiety disorders. Notably, anxiety disorders caused by substance-induced and those resulting from a general medical condition were not included.

Given that the GBD electronic databases for mental disorders are continuously updated, no additional electronic search was required for GBD 2021. However, a grey literature review and expert consultation were conducted. They included studies reporting on the incidence, prevalence, remission, duration, and increased mortality attributable to anxiety disorders. These studies had to meet the following GBD inclusion criteria: the publication year had to be 1980 or later; the assessment of ‘caseness’ required the use of clinical threshold as defined by the ICD or DSM; studies were required to include comprehensive information about their sample characteristics and methodology to facilitate a quality assessment; the samples had to be drawn from the general population, meaning samples from inpatients, patients receiving pharmaceutical treatment, case studies, veterans, or refugees were not allowed; the publication’s language was unrestricted. The detailed methodologies utilised in this systematic review are comprehensively documented in another source (2024b). Future updates of the electronic database will be conducted in the next GBD round.

The retrieved data were subjected to three rigorous gender and age separation methods: 1) Estimates were subdivided by age and gender where applicable, using the available data. For example, when studies provided prevalence for wide age categories by gender and also by specific age groups for both genders, age-specific estimates were separated by gender using the stated sex ratio and limits of uncertainty. 2) The remaining both-sex estimates in the sample were divided utilising a Meta-Regression with Bayesian priors, Regularisation, and Trimming (MR-BRT) analysis. This involved an MR-BRT network meta-analysis to derive pooled sex ratios and limits of uncertainty for each parameter by matching sex-specific estimates by location, age, and year. These refined estimates were then applied to the dataset to create separate male and female estimates. 3) Using the prevalence age pattern predicted by DisMod-MR 2.1, studies providing prevalence estimates across age groups spanning at least 25 years were divided into five-year age groups. It is important to note that the DisMod-MR model utilised to assess the age distribution did not include any previously age-split information, ensuring the integrity of the analysis.

### Data processing and disease model

Bias corrections, or crosswalks, are techniques used to adjust or align data to compensate for any inherent biases or discrepancies. Before using DisMod-MR 2.1, estimates with known biases were appropriately corrected or cross-checked by IHME. All past-year recall estimates for anxiety disorders were adjusted using a past-year recall ratio to approximate the levels they would have reached if point or past-month prevalence had been captured. Recall bias has a smaller effect on the latter prevalence period (2024b).

The epidemiological data on anxiety disorders were modelled by IHME using DisMod-MR 2.1. When outliers were detected, IHME conducted a thorough re-evaluation of the study’s quality and methodology before deciding whether to reject or include the data.

The modelling technique initially included data across all epidemiological parameters. Due to the wider global coverage of prevalence studies compared to incidence studies, the IHME opted to exclude incidence studies and instead relied on information from other parameters. It was hypothesised that neither incidence nor prevalence existed before 2 years old or after 95 years old. Expert opinions and current research on anxiety disorders supported this minimum age of onset. Based on the available data points, the maximum remission value was set at 0.2.

### Impact of COVID-19

The possible impact of the pandemic on mental health has been a source of great concern since its emergence in 2020. GBD 2021 assessed the impact of the pandemic on the burden and prevalence of anxiety disorders in 2020 and 2021. Initially, a thorough examination of the existing literature was conducted to identify research that documented the prevalence of anxiety disorders during the pandemic. This review included publications from 1 January 2020 to 29 January 2021. The inclusion criteria were closely aligned with the broader criteria used in the GBD to ensure measurement consistency (2024b). The research required the inclusion of a pre-pandemic baseline and an assessment of anxiety disorder prevalence during the pandemic. Preference was given to longitudinal studies that used representative samples from the general population. However, cross-sectional studies conducted during the pandemic were also accepted if they provided comparable pre-COVID prevalence data. Due to a lack of data using diagnostic thresholds for anxiety disorders, studies reporting on probable anxiety disorders, psychological distress, or combined anxiety and depression using validated screening tools (e.g. the General Anxiety Disorder-7 or Kessler-6) were included and adjusted for in the analysis (2024b). After completing the regular epidemiological modelling analysis, the COVID-19 adjustment was applied to the prevalence estimates for 2020 and 2021. To explore the effect of the pandemic on anxiety disorder prevalence, the prevalence statistics from the COVID-19 systematic review were analysed separately (Santomauro *et al*., 2022). The logit difference between pre-pandemic and pandemic prevalence was calculated for each set of eligible input data. The model’s adjustment to prevalence was evaluated through the implementation of two key steps, detailed in other sources (2024b).

DisMod-MR 2.1 approximated the age-, sex-, and location-specific prevalence of anxiety disorders for 2020 and 2021 based on pre-2020 prevalence data. The projected logit change from the MR-BRT model was the applied for every day of those years. After determining the mean daily prevalence for the year, the annual point prevalence estimates for 2020 and 2021 were calculated (2024b).

### Disability weight and severity distribution

The GBD disability weight survey assessments provide accessible explanations of sequelae, highlighting the severe symptoms and functional impacts. Anxiety disorders were classified by severity into asymptomatic, mild, moderate, or severe. Additional details on severity levels and associated disability weights can be found in Table S1. The years of life lost due to premature death (YLLs) and the years lived with disability (YLDs) were added together to create the DALY, a common measure used to evaluate the burden of a disorder or disease (2024b). YLDs are determined by multiplying the prevalence in each severity group by the severity-specific disability weights. Because there were no YLLs or mortalities attributable to anxiety disorders, the DALYs were equivalent to the YLDs (2024b). Uncertainty intervals (UIs) (95%) were created by performing 1,000 iterations at each computing step, incorporating uncertainty from multiple sources, such as input data, estimations of residual non-sampling error, and corrections of measurement error. The UIs consisted of the 2.5th and 97.5th percentiles of the sorted draws.

The association between the burden of anxiety disorders, as evaluated by YLDs, and SDI was investigated using smoothing splines. SDI scores range from 0 to 1, where 0 indicates the least developed and 1 indicates the most developed. These scores are derived from the total fertility rate for women under 25, the mean years of education for those over 15, and the smoothed gross domestic product per capita over the preceding 10 years. Utilising R software (V 3.5.2), we plotted the age-standardised point prevalence, incidence, and YLD rates (2024b).

To gain a comprehensive understanding of the methodology employed in this study, please refer to Supplementary File 1 provided from the GBD 2021 study (2024b).

## Results

### The Middle East and North Africa region

With an age-standardised point prevalence of 5.95 thousand (95% UI: 2.8 to 3.9) cases per 100,000 population, anxiety disorders accounted for approximately 37.8 million (30.9 to 46.2) prevalent cases in the region in 2021. The age-standardised point prevalence of anxiety disorders increased by approximately 20.3% (12 to 28.5) from 1990 to 2021 (Table 1 and Table S2). Anxiety disorders had an age-standardised incidence rate of 883.4 (722.4 to 1108.5) cases per 100,000 people in 2021, accounting for 5.7 million (4.6 to 7.1) incident cases. In comparison to 1990, the age-standardised incidence rate of anxiety disorders rose by approximately 21.3% (12.4 to 29.7) (Table 1 and Table S3). Additionally, 4.5 million YLDs (2.9 to 6.3) were attributed to anxiety disorders in 2021, with an age-standardised rate of 707.1 (469.8 to 994.9) per 100,000. Similar to the age-standardised point prevalence and age-standardised incidence rate, the YLD rates of anxiety disorders in 2021 were 20.3% (11.8 to 28.3) higher compared with 1990. (Table 1 and Table S4).

**Table 1.** Prevalence, incidence and YLDs due to anxiety disorders in 2021, and percentage change in age-standardised rates from 1990 to 2021

	Prevalence (95% UI)			Incidence (95% UI)			YLD (95% UI)		
	**Counts** **(2021)**	**ASRs** **(2021)**	**Pcs in ASRs** **1990-2021**	**Counts** **(2021)**	**ASRs** **(2021)**	**Pcs in ASRs** **1990-2021**	**Counts** **(2021)**	**ASRs** **(2021)**	**Pcs in ASRs** **1990-2021**
**North Africa and Middle East**	37799731 (30917632, 46289421)	5950.3 (4892.2, 7232)	20.3 (12, 28.5)	5702847 (4644052, 7167442)	883.4 (722.4, 1108.5)	21.3 (12.4, 29.7)	4508067 (2969187, 6368321)	707.1 (469.8, 994.9)	20.3 (11.8, 28.3)
**Afghanistan**	1780311 (1256460, 2503673)	6036.1 (4354.2, 8133.3)	21.6 (−6.5, 53.5)	303477 (212742, 426472)	901.4 (640.4, 1264.1)	24.8 (−2.7, 54.7)	212963 (129925, 320564)	708.3 (439.8, 1058)	22.1 (−5, 52.9)
**Algeria**	2405797 (1696838, 3263059)	5466.5 (3865.7, 7367.7)	13.5 (−13.6, 46.8)	363903 (255478, 506148)	812.8 (572.9, 1135.8)	14.5 (−12.8, 46.7)	286953 (176141, 418472)	651 (400.8, 949.5)	13.3 (−14.1, 47.2)
**Bahrain**	93,622 (66,063, 123860)	5720 (4016, 7508.8)	16.5 (−9.2, 49.1)	13,861 (9613, 18,860)	884.6 (622.2, 1191.8)	21.4 (−5.1, 58.2)	11,201 (6731, 16,894)	682.8 (412.7, 1045)	16.6 (−9.7, 50.2)
**Egypt**	5481791 (3911372, 7472414)	5213.9 (3730.1, 7105.7)	20.6 (−6.9, 52)	899197 (636150, 1243266)	813.5 (581.6, 1118.2)	23.2 (-5.4, 56.4)	658669 (380569, 983989)	622.3 (363.7, 915.9)	20.8 (−6.9, 53.4)
**Iran**	7288282 (6217244, 8378425)	8198.4 (7057.5, 9426.2)	21.3 (14, 28.7)	986389 (808525, 1216359)	1142 (938.6, 1401.4)	23.1 (15.5, 30.7)	864198 (587427, 1191910)	973.7 (659.1, 1345.8)	21.5 (14.1, 28.7)
**Iraq**	2460206 (1733541, 3379014)	5837.9 (4136.6, 7887)	16.8 (−10.7, 48.3)	373399 (263928, 520561)	846.8 (608.5, 1170.4)	17.9 (-8.5, 50.3)	293838 (177147, 444955)	690.9 (425, 1028.3)	17 (−10.6, 48.8)
**Jordan**	737436 (513268, 978697)	5694.7 (4016.1, 7542)	16.2 (−10.3, 47.6)	112466 (80,016, 156130)	852.5 (609.3, 1182.4)	18.3 (−8.4, 49.4)	88,575 (52,135, 137122)	679.2 (404.1, 1050.3)	16.3 (−10.6, 47.5)
**Kuwait**	256068 (181744, 346024)	5065.6 (3632.5, 6817.4)	12.1 (−12.5, 42.4)	36,403 (26,141, 50,368)	761.4 (556.4, 1056)	10.2 (−13.7, 41)	30,497 (18,804, 46,382)	604.1 (377.5, 926.5)	11.6 (−12.8, 42.7)
**Lebanon**	473053 (331020, 634186)	8274.7 (5766.6, 11,065.3)	36.1 (6.7, 72.9)	63,879 (45,411, 85,516)	1168 (824, 1570.7)	42.4 (10.8, 80.1)	55,917 (33,924, 82,475)	981 (592.8, 1442.8)	36.3 (6.2, 72.6)
**Libya**	458304 (328436, 626545)	6188.3 (4448, 8520)	22.9 (−2.6, 53.8)	65,019 (45,650, 89,924)	910.9 (645.2, 1275.3)	23.8 (−2.4, 54.4)	54,530 (33,340, 80,996)	735.5 (441.9, 1099.2)	22.3 (−3.7, 52.8)
**Morocco**	2282348 (1643839, 3088910)	5985.1 (4316.5, 8093)	23.5 (−3, 59.5)	336701 (246909, 452168)	894.6 (658.9, 1198.9)	25.5 (−2.8, 61.2)	270397 (168855, 405942)	708.9 (442.7, 1062.7)	23.3 (−3.5, 58.2)
**Oman**	271740 (189928, 370977)	5624.3 (3947.3, 7639.4)	20.6 (−7, 50.9)	42,559 (30,713, 57,926)	869.9 (624.1, 1158.1)	23.3 (−5.4, 54.3)	32,703 (19,847, 48,698)	673.1 (412.2, 1001.8)	21 (−7.1, 52.1)
**Palestine**	327024 (230094, 445128)	6302.6 (4552, 8392.6)	19 (−9.1, 53.8)	51,315 (36,397, 70,569)	921.2 (657.7, 1246.9)	22.7 (−6.9, 58.7)	39,245 (23,650, 60,192)	747.8 (457, 1112.1)	19 (−9.4, 53.5)
**Qatar**	155078 (107588, 219879)	4892.2 (3499.4, 6708.9)	10.3 (−15.2, 41.1)	23,539 (15,859, 32,553)	773.4 (547.5, 1069.2)	11.9 (−13.8, 43.7)	18,560 (11,154, 27,035)	583.1 (363, 829.7)	9.9 (−16.5, 40.7)
**Saudi Arabia**	2108231 (1495959, 2885792)	5144.1 (3714.6, 6929.1)	12.2 (−14, 43.9)	310803 (223060, 432305)	788.5 (572.3, 1076.1)	13.3 (−12.8, 44.6)	251809 (157586, 378772)	612.8 (381.2, 918.2)	12.3 (−14.1, 44.8)
**Sudan**	2497205 (1789131, 3544144)	5734.4 (4172.6, 8075.9)	18 (−10.4, 52.3)	403701 (280070, 577617)	858.7 (612.4, 1211.3)	20.3 (−7.7, 53.8)	300202 (185510, 468843)	681.9 (428.1, 1046.7)	18.3 (−10.8, 53.6)
**Syrian Arab Republic**	945251 (668283, 1288268)	6427.4 (4549.3, 8864.8)	24.4 (−5.6, 59.1)	134788 (96,450, 183583)	924 (659.9, 1258.7)	23.8 (−4.6, 57.7)	112090 (70,647, 167371)	759.8 (483.2, 1145.4)	23.5 (−6.3, 57)
**Tunisia**	826676 (598155, 1122049)	6875.4 (4987.4, 9496.7)	37.3 (6.4, 73.6)	118721 (83,853, 168901)	1025.7 (722.5, 1475)	40.8 (7.9, 80.6)	97,771 (61,489, 144542)	816.8 (506.6, 1206.5)	36.6 (5.6, 73.7)
**Turkey**	4694266 (3283843, 6411243)	5420.1 (3786.6, 7370.4)	33.5 (−4.1, 77.6)	698666 (503758, 964631)	841.3 (608.4, 1151.4)	33.4 (−1.2, 75.5)	559351 (332345, 830643)	648.1 (385.2, 958.6)	33.7 (−3.8, 77.5)
**United Arab Emirates**	519114 (358345, 711671)	5168.1 (3648.7, 7062.6)	20.6 (−9.3, 55.7)	79,861 (54,355, 114045)	835.4 (606.5, 1165.6)	23.7 (−6.4, 60.2)	61,832 (37,158, 96,015)	618.4 (374.2, 940.3)	20.7 (−9.5, 58)
**Yemen**	1702674 (1218061, 2315536)	5203 (3785.3, 6971.3)	5.5 (−18.3, 34.3)	278881 (198872, 387103)	769.1 (553.6, 1044.7)	6.7 (−18.5, 37.3)	202561 (122312, 306292)	611.7 (374.9, 903.7)	5.6 (−18.4, 34.1)

### National level

**Figure 1.**
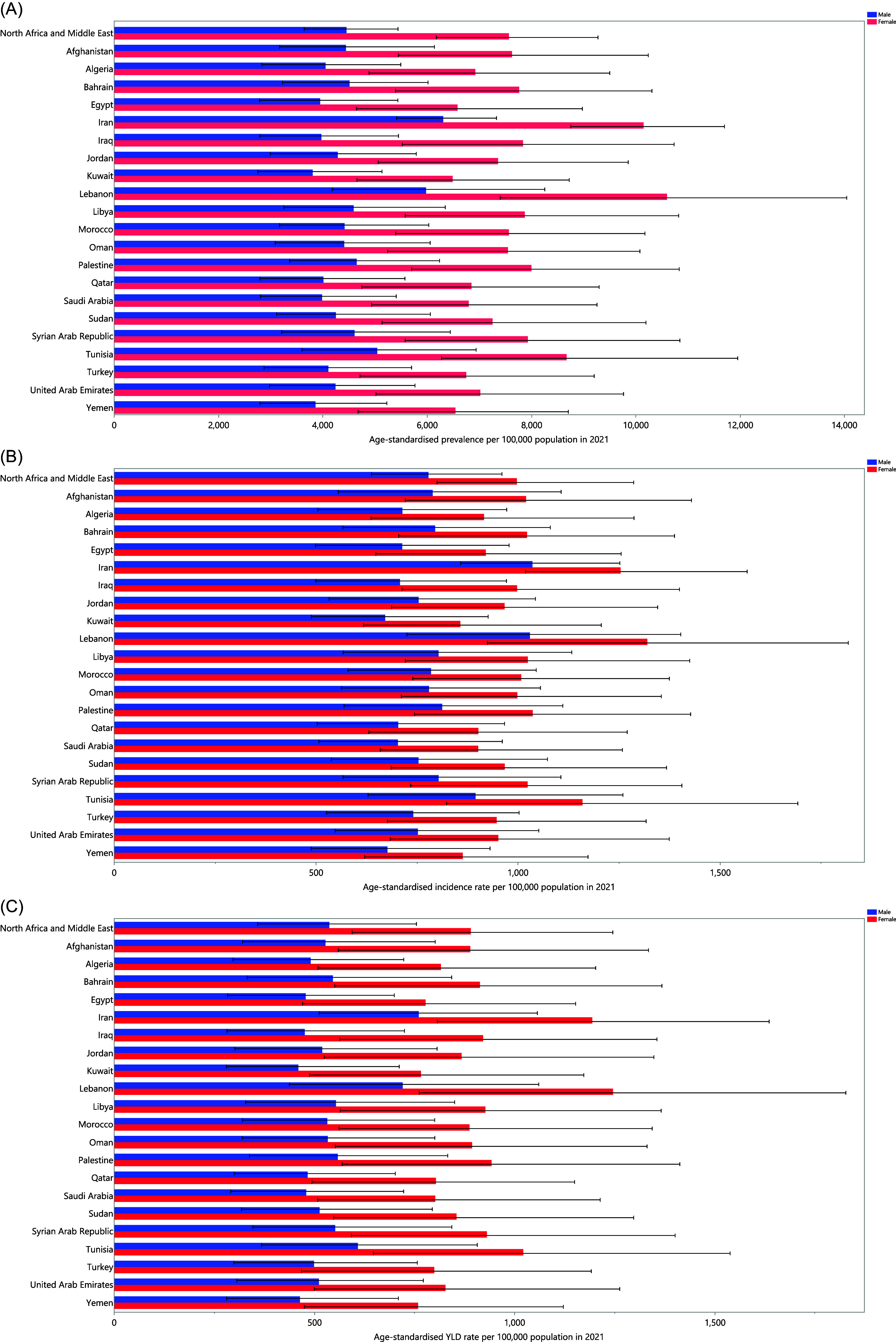
Age-standardised prevalence (*A*), incidence (*B*), and YLDs (*C*) for anxiety disorders (per 100,000 population) in the Middle East and North Africa region in 2021, by sex and country. YLD = years lived with disability. (Generated from data available from https://ghdx.healthdata.org/gbd-results-tool).

In 2021, the age-standardised point prevalence of anxiety disorders in MENA varied from 4.8 to 8.2 thousand cases per 100,000 people. Lebanon [8274.7 (5766.6 to 11,065.3)], Iran [8198.4 (7057.5 to 9426.2)], and Tunisia [6875.4 (4987.4 to 9496.7)] had the highest point prevalences, while Saudi Arabia [5144.1 (3714.6 to 6929.1)], Kuwait [5065.6 (3632.5 to 6817.4)], and Qatar [4892.2 (3499.4 to 6708.9)] had the lowest (Table S2). Fig. 1a illustrates the national age-standardised point prevalence of anxiety disorders for both genders in 2021. The age-standardised incidence rate of anxiety disorders in 2021 varied between 3.9 and 7.7 thousand cases per 100,000 people. The highest age-standardised incidence rates were observed in Lebanon [1168 (824 to 1570.7)], Iran [1142 (938.6 to 1401.4)], and Tunisia [1025.7 (722.5 to 1475)]. According to Table S3, Qatar, Yemen, and Kuwait exhibited the lowest rates, with values of 773.4 (547.5 to 1069.2), 769.1 (553.6 to 1044.7), and 761.4 (556.4 to 1056), respectively. Fig. 1b depicts the age-standardised incidence rate of anxiety disorders for both genders at the national level in 2021.

The age-standardised YLD rate for anxiety disorders varied from 583.1 to 981 cases per 100,000 people in 2021. The highest age-standardised YLD rates were observed in Lebanon [981 (592.8 to 1442.8), Iran [973.7 (659.1 to 1345.8)], and Tunisia [816.8 (506.6 to 1206.5)], while the lowest were found in Yemen [(611.7 (374.9 to 903.7)], Kuwait [604.1 (377.5 to 926.5)], and Qatar [(583.1 (363 to 829.7)] (Table S4). Fig. 1c presents the national age-standardised YLD rate of anxiety disorders for both genders in 2021.

Three MENA countries experienced a significant rise in the age-standardised point prevalence of anxiety disorders between 1990 and 2021. The countries with the biggest increases were Tunisia [37.3% (6.4 to 73.6)], Lebanon [36.1% (6.7 to 72.9)], and Iran [21.3% (14 to 28.7)]. However, the increases in other MENA countries were not statistically significant (Fig. S1 and Table S2).

Lebanon, Tunisia, and Iran exhibited the largest rises in the age-standardised incidence rate of anxiety disorders, with increments of 42.4% (10.8 to 80.1), 40.8% (7.9 to 80.6), and 23.1% (15.5 to 30.7), respectively. However, the increases in other MENA countries were not statistically significant (Fig. S2 and Table S3). In Tunisia [(36.6% (5.6 to 73.7)], Lebanon [36.3% (6.2 to 72.6)], and Iran [21.5% (14.1 to 28.7)], the age-standardised YLD rate increased during the same time period. However, the increases in other MENA countries were not statistically significant (Fig. S3 and Table S4).

### Age and sex patterns

In 2021, the regional prevalence and YLD cases of anxiety disorders in men increased up to the 10-14 age group, and then declined with increasing age. In contrast, the prevalence and YLD cases in women peaked in the 15–19 age group and subsequently declined as they aged. Furthermore, in both sexes, the regional age-standardised point prevalence and YLD rates of anxiety disorders increased up to the 15–19 age group before declining with advancing age. The regional prevalence, YLD cases, and rates were consistently higher in females than in males.

**Figure 2.**
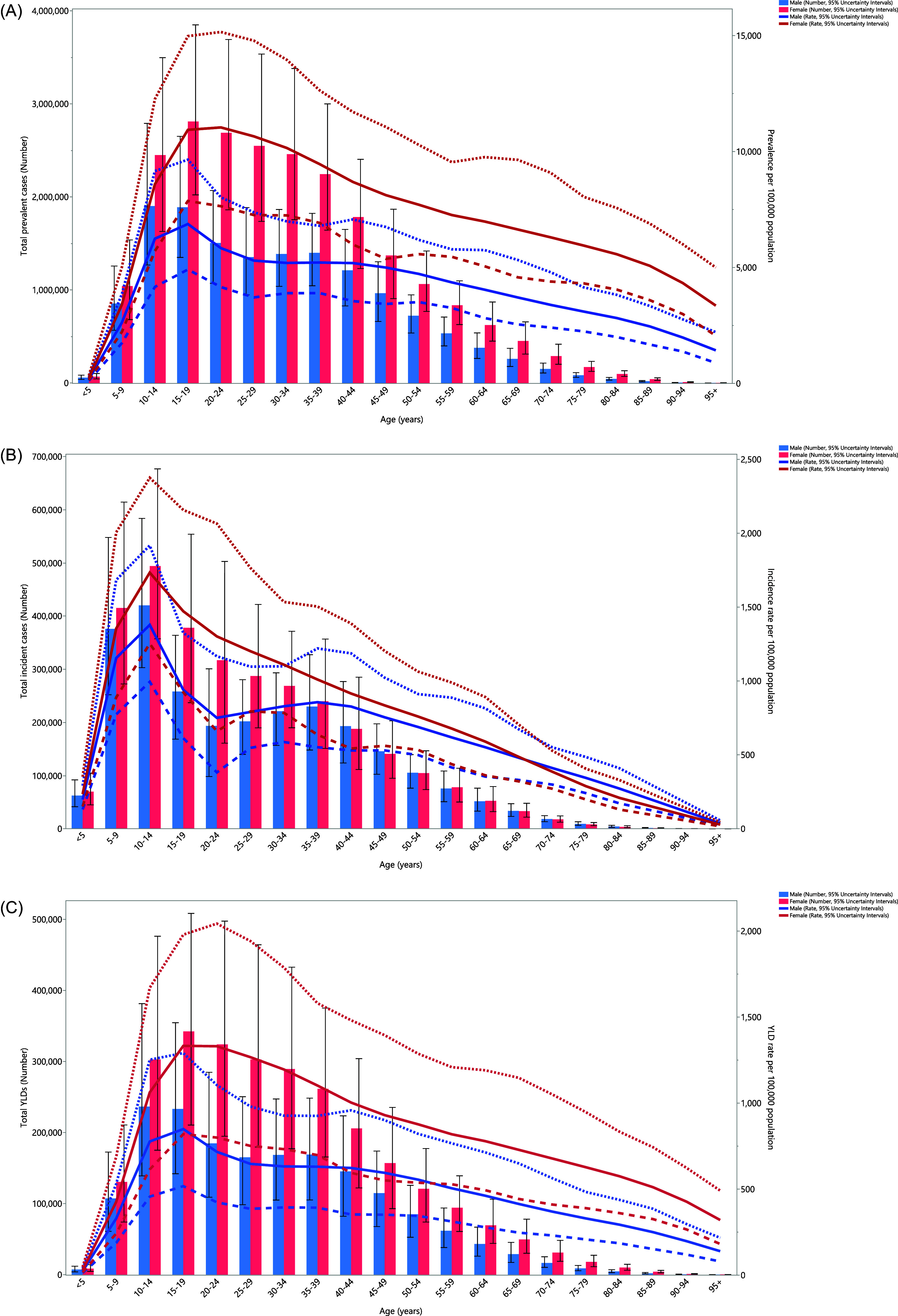
Number of prevalent cases and rate (*A*), number of incident cases and incidence rate (*B*), and the number of YLDs and YLD rate (*C*) of anxiety disorders per 100,000 population in the Middle East and North Africa region, by age in 2021; dotted and dashed lines indicate 95% upper and lower uncertainty intervals, respectively. YLD = years lived with disability. (Generated from data available from https://ghdx.healthdata.org/gbd-results-tool).

Females had a higher incidence of anxiety disorders until the 45–49 age group, after that, males had a higher incidence, except for the 55–59 age group. The regional incidence of anxiety disorders increased significantly in both sexes up to the 10–14 age range, then decreased with advancing age. Moreover, the age-standardised incidence rate of anxiety disorders in the region exhibited a significant increase up to the 10–14 age range, and then declined as age advanced in both males and females. The regional age-standardised incidence rate of anxiety disorders was higher in females up to the 65–69 age group, after which males exhibited higher rates (Fig. 2a–c).

In 2021, the MENA/Global YLD rate ratio for anxiety disorders was highest in the under-5 age group, with a ratio of 2.1 for males and 1.7 for females. This ratio decreased with advancing age, becoming equal to the global rate in the 65–74 age groups for males. The remaining age groups had lower rates than the global average, except for the 95+ age group, which was equal to the global rate. Similarly, in females, this ratio decreased with age, becoming the same as the global rate in the 70–74 and older age groups, except for the 95+ age range, which was below the global average.

**Figure 3.**
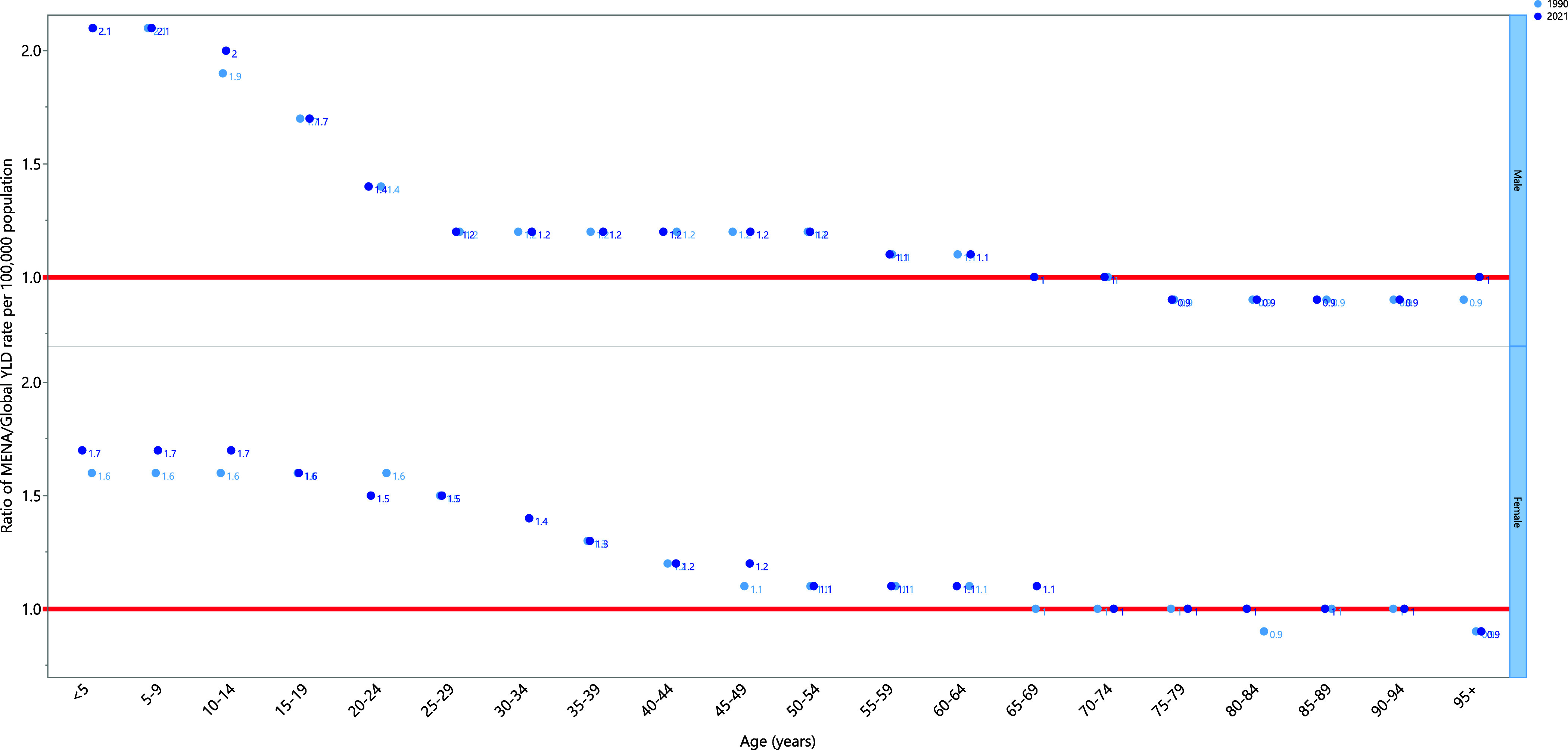
Ratio of the Middle East and North Africa region to the global YLD rate of anxiety disorders by age and sex in 1990 and 2021. YLD = years lived with disability. (Generated from data available from https://ghdx.healthdata.org/gbd-results-tool).

Compared to 1990, there were no differences in YLD rates among age groups for males, except for the 10–14 and 95+ age groups, which had higher rates in 2021 than in 1990. In 2021, females in the 0–14, 45–49, 65–69, and 80–84 age groups had higher rates compared to 1990. Additionally, in females, the YLD rate for all age groups remained consistent with 1990 levels, except for the 20–24 age group, which had a lower rate (Fig. 3).

### Socio-demographic index (SDI) and anxiety disorders

**Figure 4.**
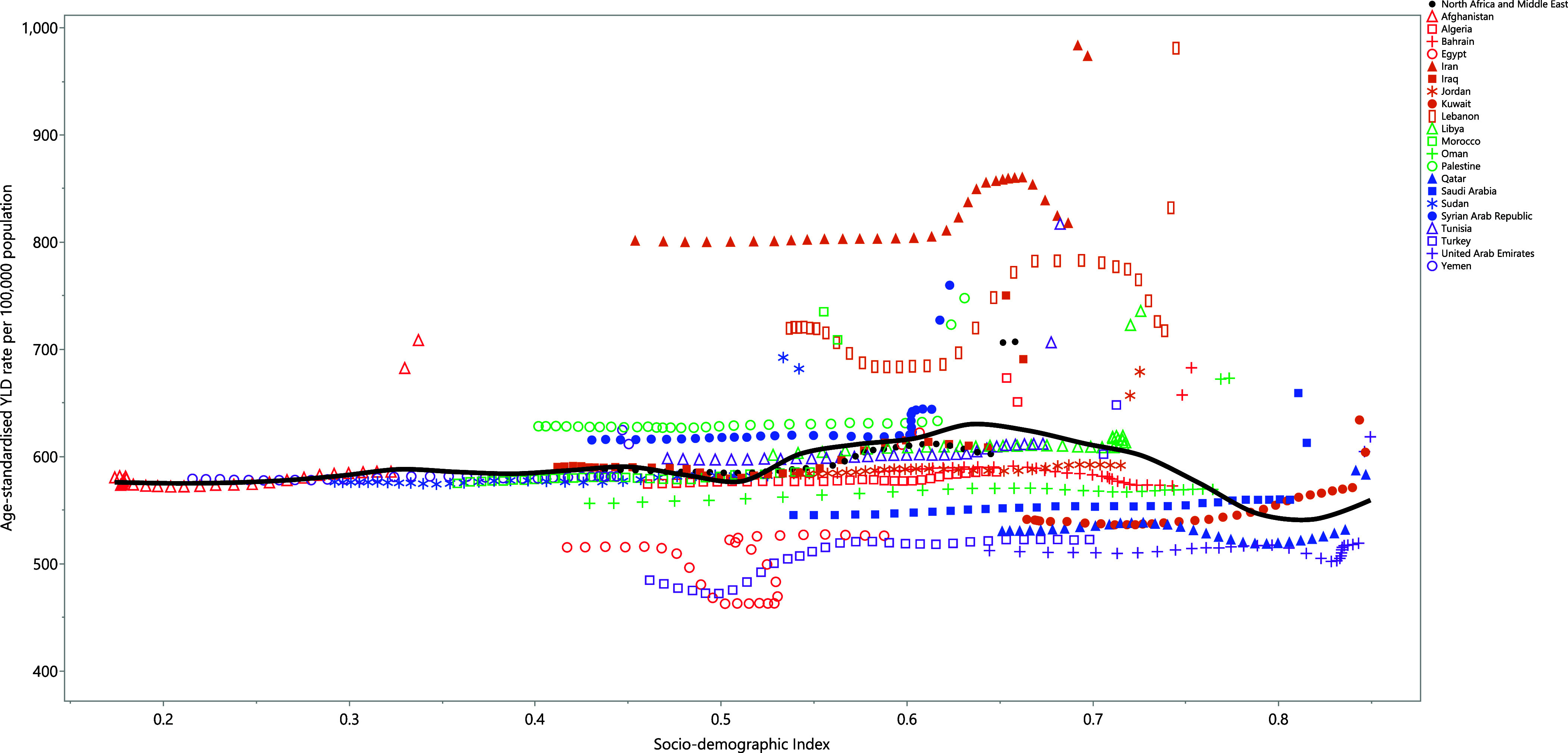
Age-standardised YLD rates of anxiety disorders for the 21 MENA countries by SDI, 1990–2021; expected values based on the sociodemographic index and disease rates in all locations are shown as the black line. Each point shows the observed age-standardised YLD rate for each country during 1990–2021. YLD = years lived with disability. SDI = sociodemographic index (Generated from data available from https://ghdx.healthdata.org/gbd-results-tool).

The burden of anxiety disorders did not have a linear relationship with SDI. Nevertheless, a small increase in the burden of anxiety disorders was noted with increasing development from an SDI level of 0.5 to 0.65. It then decreased slightly up to an SDI level of 0.7, after which this decreasing trend became more pronounced, reaching the lowest YLD rate at an SDI of 0.8, after which it increased with increasing SDI level. Lebanon, Iran, Palestine, and the Syrian Arab Republic had higher than expected burdens, while Egypt, Turkey, the United Arab Emirates, Qatar, Kuwait, Saudi Arabia, Oman, Bahrain, and Jordan had lower than expected burdens (Fig. 4).

## Discussion

This research presented current data on the incidence, prevalence, YLDs, and age-standardised rates of anxiety disorders for the 21 MENA nations from 1990 to 2021. In 2021, anxiety disorders accounted for 37.8 million prevalent cases, 5.7 million incident cases, and 4.5 million YLDs. Furthermore, the incidence rate increased by 21.3%, and the age-standardised prevalence and YLD rates have risen by 20.3% since 1990. The age-standardised prevalence, incidence, and YLD rates were highest in Lebanon, Iran, and Tunisia. Conversely, the lowest age-standardised YLD rates were found in Yemen, Kuwait, and Qatar, while the lowest age-standardised prevalence rates were observed in Saudi Arabia, Kuwait, and Qatar. The lowest age-standardised incidence rates were seen in Qatar, Yemen, and Kuwait. Furthermore, the burden related to anxiety disorders increased up to the 15–19 age group, after which it declined with age, with women bearing a consistently greater burden. Additionally, the MENA region exhibited higher YLD rates due to anxiety disorders across most age groups when compared to the global average. Lastly, the analysis revealed no clear association between SDI and the burden of anxiety disorders.

A number of researchers have rigorously investigated the global burden of anxiety disorders (Xiong *et al*., 2022, Baxter *et al*., 2014, Yang *et al*., 2021). In addition, several national and international studies have offered estimates of the burden of mental disorders, including anxiety disorders (Baxter *et al*., 2016, Chen *et al*., 2024, Charara *et al*., 2017, Iovu & Breaz, 2019, Ciobanu *et al*., 2018, Bonadiman *et al*., 2017, Rehm & Shield, 2019, Charara *et al.,* 2017, Castelpietra *et al*., 2022, Ferrari *et al*., 2022, Hong *et al*., 2022, Whiteford *et al*., 2015). In contrast, only one previous study has documented the burden of mental disorders in the MENA region (Effatpanah *et al*., 2024). However, this study is now outdated, particularly due to the fact that the recent COVID-19 pandemic significantly affected mental health.

In 2019, global age-standardised rates of anxiety disorders were estimated to be 3779.5, 585, and 360 per 100,000, for prevalence, incidence, and DALYs, respectively (Yang *et al*., 2021, 2022). In MENA, these rates were 5135.7, 783, and 492 per 100000, respectively (Yang *et al*., 2021, Ferrari *et al.,* 2022). According to the current study’s results, the MENA region had a higher burden of anxiety disorders, although the estimated prevalence and incidence rates are quite similar to previous studies. This may have been influenced by the 2020 COVID-19 pandemic, which increased the burden of anxiety disorders. A previously released study reported that the COVID-19 pandemic caused an extra 1664.8 cases of anxiety disorders per 100,000 people in 2020, representing a 32.4% increase (2021). In the MENA region, the estimated prevalence of anxiety disorders was 5148.9 per 100,000 population prior to the pandemic. After adjusting for the pandemic, the prevalence in 2020 was 6813.6 per 100,000 (2021). During the pandemic, quarantine measures played a significant role in contributing to the burden of COVID-19–related anxiety disorders. It has been demonstrated that quarantine has a major psychological impact, especially on hospital employees and those who are impacted. A study revealed that quarantine was the most significant predictor of acute stress symptoms immediately following the event, with personnel experiencing weariness, alienation, worry, irritability, sleeplessness, decreased job performance, and even contemplating resignation (Bai *et al*., 2004). Quarantine measures were associated with persistent mental health consequences, with hospital staff showing elevated rates of depression and PTSD symptoms that endured for up to three years later (Wu *et al*., 2009). Comparative analyses revealed quarantined children had post-traumatic stress scores that were four times higher than those of non-quarantined peers. Among parents, 28% of those quarantined met the diagnostic criteria for trauma-related mental health disorders versus 6% of non-quarantined controls (Sprang & Silman, 2013).

During quarantine, insufficient access to essential resources such as food, water, clothes, and shelter was a major cause of dissatisfaction and correlated with heightened anxiety and anger, enduring for months after the quarantine period (Blendon *et al*., 2004, Wilken *et al*., 2017, Jeong *et al*., 2016). Moreover, interruptions in standard medical care exacerbated these challenges (Blendon *et al*., 2004). A key obstacle was the financial cost of quarantine, since those who were unable to work experienced severe socioeconomic hardship (Pellecchia *et al*., 2015). Long after the quarantine was over, this financial burden continued to be a risk factor for psychiatric problems, showing up as increased anxiety and anger (Mihashi *et al*., 2009, Jeong *et al*., 2016). Individuals with lower household incomes had elevated levels of post-traumatic stress and depressive symptoms, possibly attributable to their increased susceptibility to income loss (Hawryluck *et al*., 2004). Targeted economic interventions are needed to mitigate the mental health burden of quarantine, particularly through financial reimbursements and structured assistance programmes for low-income households (Brooks *et al*., 2020).

The stigma attached to quarantine was another persistent problem; those who were placed under quarantine frequently experienced rejection and social exclusion (Bai et al., 2004, Brooks *et al*., 2020). One of the most important steps in reducing stigma is to educate the public about the illness and the need for quarantine, as well as to give schools and workplaces relevant information (Brooks *et al*., 2020). These findings underscore the extensive and enduring mental health effects of quarantine restrictions during the COVID-19 outbreak.

Additionally, consistent with our findings, extensive research has shown that women experience higher prevalence, incidence, and YLD rates of anxiety disorders compared to men (Yang *et al*., 2021, Whiteford *et al*., 2015, Hong *et al*., 2022, Baxter *et al*., 2014, Kumar *et al*., 2024, McLean *et al*., 2011). According to McLean *et al*., one in three women will meet the criteria for an anxiety disorder at some point in their lives, compared to 22% of men (McLean *et al*., 2011). Their study found that women had lifetime and past-year rates of anxiety disorder that were 1.5 to 2 times higher than those of men (McLean *et al*., 2011). Gender socialisation plays a role in shaping the psychosocial traits that serve as risk factors for women and protective factors for men in the initiation and progression of anxiety (Leach *et al*., 2008, Seedat *et al*., 2009, Stoyanova & Hope, 2012, Zalta & Chambless, 2012, McLean & Anderson, 2009). In addition, anxiety issues may be exacerbated by the inferior living conditions experienced by women. Studies have indicated that one of the main obstacles to improving women’s health is the social environment, which includes the market economy, beauty standards, sexual division of labour, and environmental degradation (Sánchez, 2018). Socioeconomic crises, such as the one following the COVID-19 pandemic, have the potential to exacerbate these issues. As previously mentioned, the COVID-19 pandemic led to an increase in the prevalence and disease burden of anxiety disorders, with women experiencing this rise at a significantly higher rate than men (2021). One plausible explanation is that women are disproportionately affected by adverse social and economic effects (Hupkau & Petrongolo, 2020, Women, 2020, Wenham *et al*., 2020, Burki, 2020). They were more likely to assume additional caregiving and household responsibilities due to sick family members or school closures (Women, 2020). Women faced greater financial hardship during the pandemic, primarily due to lower wages, smaller financial reserves, and more unstable employment compared to men (Women, 2020, Wenham *et al*., 2020, Burki, 2020). Furthermore, women faced a higher risk of becoming targets of domestic violence, a problem that intensified during lockdowns and stay-at-home mandates (Piquero *et al*., 2021, Arenas-Arroyo *et al*., 2021). The financial crisis of 2009 and other socioeconomic strains have been shown to significantly influence the exacerbation of mental illnesses, including anxiety disorders (Frasquilho *et al*., 2016, Economou *et al*., 2013). Hence, social safety nets and support programmes that help those facing financial hardships can be implemented by policymakers. The reduction of economic stressors that contribute to anxiety disorders could be achieved by implementing these measures, which could also promote mental well-being in the community.

Genetic and neurobiological factors, alongside psychosocial factors, have been explored as potential reasons for the increased incidence of anxiety disorders among women. Fluctuations in sex hormones that affect women’s anxiety (Li & Graham, 2017, Hodes & Epperson, 2019, Donner & Lowry, 2013, Day & Stevenson, 2020, McLean *et al*., 2011, Lungu *et al*., 2015), genetic factors (Merikangas & Almasy, 2020, Donner & Lowry, 2013, Brivio *et al*., 2020, Palma-Gudiel *et al*., 2019, Burton *et al*., 2015, Ask *et al*., 2014), and differences in brain structures related to emotional regulation – which influence how negative information is processed (Cerasa *et al*., 2014, Gardener *et al*., 2013, Kaczkurkin *et al*., 2019) – are all considered potential contributors to this gender disparity.

Nevertheless, although men appear to exhibit lower levels of anxiety, some experts warn that this interpretation may be biased. According to a recent systematic review on anxiety in males, men generally prefer to rely on themselves rather than seek assistance when managing anxiety (Fisher *et al*., 2021). This tendency aligns with masculine gender norms. Men’s anxiety has also been linked to other potential risk factors, such as mistrust of receiving assistance, a lack of awareness of anxiety, and limited means for seeking treatment (Clark *et al*., 2018).

The MENA region had higher age-standardised prevalence, incidence, and YLD rates of anxiety disorders than the global averages, as previously mentioned. This discrepancy can be attributed to several factors. Firstly, varying attitudes towards mental illness across countries can foster a reluctance to recognise symptoms of psychiatric disorders (Andrade *et al*., 2014). This challenge is especially acute for anxiety disorders, as their symptoms can be profoundly embarrassing (Sorsdahl *et al*., 2012). Furthermore, mental illnesses may manifest and be experienced differently in various settings, and survey questions might not adequately account for this heterogeneity (Lewis-Fernández *et al*., 2010). In specific contexts where emotional reactions like anxiety play an adaptive role, it can be particularly difficult to differentiate between a pathological disorder and normal distress (Nesse & Stein, 2012). Additionally, the use of diagnostic interviews in epidemiological surveys can be undermined by poorly formulated questions, potentially introducing substantial biases that vary across nations (Stein *et al*., 2017). Moreover, there may be significant variations in prevalence estimates due to country-specific differences in survey field settings, such as sampling, quality control and the level of interviewer training. Finally, as risk and resilience factors vary significantly between geographic regions, there may be true variations in the frequency of mental illnesses among nations (Stein *et al*., 2017). In order to address this issue, the establishment of a regional mental health research and surveillance network across MENA countries would represent a transformative approach to addressing this growing public health challenge. This network would deliver essential data, encourage evidence-based interventions, and enhance collaboration among nations, eventually improving mental health outcomes for individuals in the region.

Compelling global evidence establishes adverse childhood experiences – particularly sexual abuse, bullying victimisation, and exposure to violence – as significant risk factors for anxiety disorders across sexes (Bauer *et al*., 2006, Bell *et al*., 2012, Blanco *et al*., 2014). In the Middle East, the estimated prevalence of intimate partner abuse is approximately 26.3%, with the most common form being psychological abuse (Moshtagh *et al*., 2023). Furthermore, MENA is the region with the highest rates of violence and armed conflict in the world (Sørli *et al*., 2005). It is also among the three regions with the highest burden of mental disorders, including anxiety disorder, linked to bullying victimisation (Hong *et al*., 2022). These findings may partially explain the high burden of anxiety disorders in MENA.

Compared to other MENA nations, Lebanon, Tunisia, and Iran had significantly higher age-standardised incidence, prevalence, and YLD rates for anxiety disorders in 2021. This finding aligns with earlier research on the burden of mental disorders in the region, which identified Palestine, Iran, and Lebanon as having the highest rates (Effatpanah *et al*., 2024). Several explanations exist for these elevated rates. For instance, as previously mentioned, childhood sexual abuse is a factor that influences anxiety disorders. However, regional level research on the prevalence of child sexual abuse is lacking. Studies conducted in Lebanon have shown that the incidence of such abuse is between 17% and 24%, which is high (Usta & Farver, 2010, El Khoury *et al*., 2021). The socioeconomic crises in these countries are another reason for these high rates. For instance, since 2019, Lebanon has seen a devastating economic collapse characterised by hyperinflation, currency devaluation, and rampant unemployment, resulting in most people living in poverty (Guеchаti & Chаmi, 2022, Youssef, 2020, Daher, 2022). The economic decline has resulted in people and families struggling to fulfil fundamental necessities, exacerbating emotions of fear and pessimism that lead to anxiety disorders. An atmosphere of constant tension is produced by the financial burden combined with sporadic fuel, power, and healthcare shortages (Guеchаti & Chаmi, 2022, Youssef, 2020, Daher, 2022). In Tunisia, inflation, deteriorating government services, and ongoing unemployment – especially among youth – are key contributors to rising anxiety levels (Matta *et al*., 2019, Achy, 2011, Ayadi & Mattoussi, 2014). The population’s anxiety has been exacerbated by these economic stressors, as well as public discontent over unfulfilled assurances of prosperity following the Arab Spring (Matta *et al*., 2019, Achy, 2011, Ayadi & Mattoussi, 2014).

Furthermore, Iran is the world’s second most sanctioned nation, after Russia (Forbes.ge & Forbes.ge, 2024). The sanctions imposed on the country have significantly impacted both Iran’s economy and the mental health of its people (Aloosh *et al*., 2019). The limitations on access to essential goods, healthcare, and services have led to widespread anxiety, helplessness, and elevated stress levels among Iranians. Inflation and job losses caused by the economic burden exacerbate feelings of fear and hopelessness in families and communities (Aloosh *et al*., 2019). There is substantial evidence that unemployment, another consequence of sanctions (Aloosh *et al*., 2019), increases the likelihood of developing and progressing psychiatric problems, particularly anxiety and depression disorders (Zhang & Bhavsar, 2013, Paul & Moser, 2009). However, reverse causation is also possible, whereby existing psychiatric conditions may increase the risk of unemployment. For instance, Mojtabi et al., demonstrated that individuals with a mental illness were more likely to experience future unemployment (Mojtabai *et al*., 2015). Political unrest, unstable economies, and cultural elements are all closely linked to mental health issues in high-burden areas like the MENA region. Resolving these issues necessitates focused, interdisciplinary strategies that integrate health, education, and social support frameworks. For the purpose of creating strong and culturally aware care systems, research on the long-term psychological effects of displacement and conflict is especially important. Cooperative initiatives across governments, NGOs, and international organisations are crucial for establishing sustainable mental health infrastructure, providing both enduring remedies and avenues for resilience and hope for communities experiencing chronic stress.

Saudi Arabia, Kuwait, and Qatar recorded the lowest age-standardised prevalence rates. A study conducted between 2014 and 2016 in Saudi Arabia assessed the lifetime prevalence of mental disorders among youth and adolescents (Altwaijri *et al*., 2023). Their study found that the lifetime prevalence of anxiety disorders was approximately 26.9%, which is significantly higher than our findings (Altwaijri *et al*., 2023). This discrepancy may be attributed to the Saudi study’s focus on a limited participant population of young adults and adolescents (Altwaijri *et al*., 2023). It is widely recognised that younger individuals exhibit a higher incidence of anxiety disorders. In a study by Kroenke et al., 19.5% of the 965 randomly sampled patients attending clinics in the US were found to have at least one anxiety disorder (29). Their study collected data using the PHQ-15 somatic symptom scale, 10-item anxiety subscale from the Hopkins Symptom Checklist, Medical Outcomes Study Short Form-20 (SF-20), 3-item version of the Social Phobia Inventory (Mini-SPIN), 5-item PHQ panic module, PHQ-8 depression scale, and GAD-7 (29). The higher prevalence reported in their study, compared to ours, may be due to their non-random sampling of patients from certain primary care clinics, which may have inflated the frequency of anxiety disorders due to the over-representation among frequent clinic attendees (29).

Research conducted in the United States has demonstrated that mental and functional health decline with poverty and low socioeconomic status (Charara *et al*., 2016, Strine *et al*., 2006, Li *et al*., 2008, Li *et al*., 2009, Strine *et al*., 2004). Higher-income nations tend to have more stable lifestyles than lower-income nations, which enables their citizens to better address their mental health needs during difficult or emergency situations (Charara *et al*., 2017). This stability could explain the lower rates of anxiety observed in the region’s countries with a higher SDI. The higher-than-expected burdens of anxiety disorders in some countries, even with their high SDI scores, could be due to the stigma-induced under-reporting, which can conceal the true extent of these disorders. Moreover, mental health burdens may be excessively affected by factors that are not directly included in the SDI, like conflict, displacement, economic instability, or climate change. This is particularly evident in certain MENA countries, where these stressors are prevalent and may exacerbate the mental health challenges faced by the population.

Our findings indicate that the incidence of anxiety disorders increased between 1990 and 2021 in most age groups, which is consistent with previous research (Vasiliadis *et al*., 2013, Yang *et al*., 2021). Additionally, the incidence rate peaked in the 10 to 14 age range. This could be linked to various factors such as low socioeconomic status, childhood abuse, corporal punishment, an overly protective or harsh parenting style, increased self-consciousness, and increased opposition to parents (Vachon *et al*., 2015, Clauss & Blackford, 2012, Moreno-Peral *et al*., 2014, Beesdo-Baum & Knappe, 2012). It is important to recognise that many anxiety disorders begin early, often serving as predictors of later psychopathology. These disorders typically onset in childhood and adolescence and tend to decline with age. Consequently, identifying at-risk individuals and implementing interventions during early life stages are crucial considerations for effective treatment, as with increasing age, rigid cognitive and behavioural patterns become ingrained and often the failure to respond to treatment stems from these entrenched behaviour patterns, which are challenging to reverse (Baxter *et al*., 2014, Craske & Stein, 2016, Grenier *et al*., 2019, Solmi *et al*., 2022, Donovan & Spence, 2000, Craske & Zucker, 2001). Moreover, mental health treatment services are frequently inaccessible or unavailable to many young people with mental health disorders. This demographic can be reached in a natural and accessible way by using the school system as the setting for prevention programmes (Gulliver *et al*., 2012). Therefore, the implementation of mental health education targeted at certain demographics, such as school students, can be cost-effective. Additionally, there is a promising potential for advancing and refining multi-channel interventions and treatments, including mental health mobile applications, internet- and telephone-based helplines, and film-based education (Holmes *et al*., 2018, Kang *et al*., 2021, White *et al*., 2022, Goodwin *et al*., 2021).

The aetiology of anxiety disorders remains a topic of debate. An overprotective parenting style, low socioeconomic status, and a parental history of mental illness are some characteristics that are thought to be linked to an elevated risk of anxiety disorders (Beesdo-Baum & Knappe, 2012, Moreno-Peral *et al*., 2014). Research in genetic epidemiology indicates that anxiety disorders display a moderate level of familial aggregation, with heritability estimated at 30–50% (Beesdo-Baum & Knappe, 2012). Moreover, there is strong evidence linking bullying victimisation to anxiety (Jadambaa *et al*., 2019). Notably, the prevalence of school bullying has been increasing, becoming a widespread issue that affects the physical and mental well-being of children and adolescents (Juvonen & Graham, 2014).

With population growth, there will likely be an increased demand for healthcare services. Furthermore, anxiety disorders can lead to significant waste of medical resources and impose a substantial burden on society and the economy, as they often develop into chronic conditions and recur in a fluctuating pattern throughout life if left untreated (Kessler *et al*., 2010). Early prevention is expected to mitigate the functional deficits associated with anxiety disorders, making it highly cost-effective (Baxter *et al*., 2014). This is particularly important in low- and middle-income nations, where providing appropriate care and treatment to the expanding population is critical (Hudson, 2005). Robust intervention methods exist for mental disorders, providing significant potential to substantially mitigate the burden these conditions impose (Reichenberg & Seligman, 2016). These treatments may decrease the risk of death, improve recovery chances, and lower symptom intensity (Belvederi Murri *et al*., 2018, Fusar-Poli *et al*., 2017). Unfortunately, significant barriers to accessing these services exist worldwide, and there is insufficient funding to support their expansion. Moreover, obstacles to care include the stigma attached to mental health conditions and the belief that one does not need treatment (Patel *et al*., 2016, Coombs *et al*., 2021).

Policymakers should urgently address the growing burden of mental disorders in the MENA region by ensuring the provision of sufficient facilities for the treatment and care of people with conditions like anxiety disorders. This is particularly crucial given the region’s inadequate infrastructure, limited services, and gaps in insurance coverage. Resolving these problems by increasing the number of mental health professionals, providing telehealth services, and subsidising mental health treatments could reduce the burden of anxiety disorders in the region (Nawaz *et al*., 2023, Pocock, 2017). Moreover, raising awareness of mental health issues in the region can help reduce stigma and encourage more people with mental health issues to seek healthcare services, considering that stigma plays a major role in creating barriers in the MENA region (Pocock, 2017, Sewilam *et al*., 2015). Furthermore, given the rate of bullying in MENA is higher than in most other regions, it is critical to address factors that increase the risk of bullying victimisation (such as social inequality, gender norms, bullying culture, and cultural conflicts) and enhance those that protect against bullying victimisation (such as parental support, school-based supportive teams, children’s rights empowerment, and an anti-bullying culture) (Hong *et al*., 2022). Successive interventions are required to prevent bullying victimisation and support victims’ mental health. Effective strategies include promoting supportive legislation and policy frameworks, minimising cultural tensions by establishing safe and welcoming environments at home and in schools (Hatzenbuehler *et al*., 2015), and reaffirming strong commitments to children’s rights and empowerment (Zambuto *et al*., 2020).

In light of the substantial and frequently neglected burden of anxiety disorders in the MENA region, it is essential to urgently strengthen national and regional mental health surveillance systems. Effective surveillance, especially the systematic gathering and analysis of data disaggregated by age, gender, and other sociodemographic factors, is crucial for monitoring epidemiological trends and evaluating the real-world impact of policy interventions. Simultaneously, emphasis should be placed on integrating evidence-based screening, prevention, and treatment approaches for anxiety disorders into the primary healthcare system. In order to improve early detection and intervention, reduce stigma, and ensure equitable access to mental health services – particularly in marginalised and conflict-affected communities – this integration must be paired with comprehensive, culturally sensitive training for primary care providers.

Additionally, it is imperative to create and execute psychoeducational and preventive initiatives that are specifically designed for adolescents and other high-risk groups, such as those impacted by conflict and forced displacement. Social determinants significantly influence mental health outcomes in the region. Therefore, collaborative multi-sectoral approaches that include education, social welfare, and the justice system are essential for addressing risk factors like violence, economic instability, and displacement. Public awareness campaigns that are culturally appropriate are also necessary to enhance mental health literacy, mitigate widespread stigma, and promote prompt assistance. Increasing investment in the training, retention, and ongoing professional development of mental health specialists, particularly in managing anxiety and trauma-related conditions, is essential for establishing a resilient and responsive mental health workforce in the MENA context.

Given all these facts, anxiety disorders represent a multidimensional burden, spanning medical, social, and economic elements. Thus, promoting interdisciplinary approaches that unite social workers, educators, legislators, and healthcare professionals is essential. This kind of cooperation can help develop comprehensive treatment plans that address the underlying causes of anxiety disorders as well as their symptoms.

### Strengths and limitations

The present research provides the most recent and comprehensive assessment of the burden of anxiety disorders in MENA, and the countries within the region, from 1990 to 2021. However, this study is not without limitations. First, data only from high-income countries were used to account for the increased burden of anxiety disorders caused by the COVID-19 pandemic (2024b). The model’s location-specific predictions relied upon two COVID-19 indicators: human mobility and predicted daily COVID-19 mortality (2024b). Due to the limited number of diagnostic mental health studies conducted during the pandemic, our analyses relied on information from symptom scales identifying probable cases of anxiety disorders (2024b). Furthermore, besides the COVID-19 data, the overall reliance was on modelled estimates and proxy indicators, often derived from high-income country data, because locally collected, diagnostic epidemiological data are scarce in many MENA countries. This scarcity is particularly pronounced in conflict-affected settings such as Syria, Yemen, and Lebanon, where compromised data collection and reporting may significantly affect the reliability and representativeness of the findings.

Second, only epidemiological estimates that included at least three anxiety disorders were considered in GBD 2021 for the outcome labelled as ‘any’ or ‘total’ anxiety disorders (2024b). The aggregation of multiple distinct anxiety disorders into a single category is another limitation of this study. This overgeneralisation restricts the clinical and practical utility of the findings for designing targeted policy or intervention strategies.

Third, there were a significant number of locations that lacked robust raw data (2024b). Fourth, measurement error in our epidemiological estimates makes it challenging to quantify and eliminate all bias (2024b). Despite enhancements to the methodology for addressing known sources of bias, the availability of data points remains limited, constraining the ability to make informed adjustments in certain instances. Fifth, the aggregation of all anxiety disorders into only one category hinders our capacity to comprehend the unique burden associated with each individual disorder. This method could obscure the differences in prevalence, severity, and effects that certain anxiety disorders – such as panic disorder, social anxiety disorder, and GAD – may have across various populations. Public health policies may become biased as a result of this constraint, as treatments created using aggregate data might not sufficiently address the particular needs of individuals with specific anxiety disorders. Notwithstanding these constraints, the study offers significant insights into the comprehensive public health implications of anxiety disorders, which can guide overarching mental health policy and resource-allocation decisions. Sixth, potential limitations in data quality exist in conflict-affected regions such as Syria, Yemen, and Lebanon. In these areas, data collection may be incomplete or biased due to ongoing violence, restricted access, and political instability. Humanitarian organisations and researchers face substantial challenges in obtaining reliable data, as conflict can disrupt communication networks and displace populations, leading to information gaps. Lastly, there is a significant gap in research regarding the risk factors for mental disorders, which limits their use as predictive variables in epidemiological models (2024b).

## Conclusions

Since 1990, the MENA region has experienced a rise in the burden of anxiety disorders. These disorders remain a significant public health concern for both the individuals concerned and their communities, given their high prevalence and substantial economic and epidemiological impact. Our study’s findings underscore the urgent need for all MENA nations to acquire current health statistics and implement enhanced policies and regulations to facilitate the early diagnosis and effective treatment of anxiety disorders.

## Supporting information

Aletaha et al. supplementary material Aletaha et al. supplementary material

## Data Availability

This study is based on publicly available data and solely reflects the opinion of its authors and not that of the Institute for Health Metrics and Evaluation. The data used for these analyses are all publicly available at https://ghdx.healthdata.org/gbd-results-tool.

## Supplementary material

Supplementary movies are available at https://doi.org/10.1017/neu.2025.10023.
