# Supplementary material for: Anxiety disorders in the Middle East and North Africa region; 1990 to 2021
Source: Acta Neuropsychiatr. 2025 Jun 30;37:e75. doi: 10.1017/neu.2025.10023 (PMC13130386; doi:10.1017/neu.2025.10023)
Supplement: Aletaha et al. supplementary material [file S0924270825100239sup001.docx]

# **Supplementary Material**

| **Table S1: Sequelae for anxiety disorders and their associated disability weights in the GBD 2021 study** | | |
| --- | --- | --- |
| **Severity**  **level** | **Lay description** | **Disability weight**  **(95% CI)** |
| Mild | Feels mildly anxious and worried, which makes it slightly difficult to concentrate, remember things, and sleep. The  person tires easily but is able to perform daily activities. | 0.03  (0.018–0.046)) |
| Moderate | Feels anxious and worried, which makes it difficult to concentrate, remember things, and sleep. The person tires  easily and finds it difficult to perform daily activities. | 0.133  (0.091–0.186)) |
| Severe | Constantly feels very anxious and worried, which makes it difficult to concentrate, remember things, and sleep. The  person has lost pleasure in life and thinks about suicide. | 0.523  (0.362–0.677) |
| **GBD: Global Burden of Disease; CI: Confidence interval** | | |

| **Table S2: Prevalence of anxiety disorders in 1990 and 2021 and the percentage change in the age-standardised rates (ASRs) per 100,000 in the Middle East North and Africa region**  **(Generated from data available from http://ghdx.healthdata.org/gbd-results-tool)** | | | | | |
| --- | --- | --- | --- | --- | --- |
|  | **1990** | | **2021** | | **Percentage change in ASRs per 100,000** |
|  | **No (95% UI)** | **ASRs per 100,000 (95% UI)** | **No (95% UI)** | **ASRs per 100,000 (95% UI)** |  |
| **North Africa and Middle East** | **15881329 (13275924 , 19086607)** | **4945.4 (4195.9 , 5812.1)** | **37799731 (30917632 , 46289421)** | **5950.3 (4892.2 , 7232)** | **20.3 (12 , 28.5)** |
| **Afghanistan** | **446420 (358287 , 558909)** | **4963.8 (4024.7 , 6114.8)** | **1780311 (1256460 , 2503673)** | **6036.1 (4354.2 , 8133.3)** | **21.6 (-6.5 , 53.5)** |
| **Algeria** | **1154354 (906541 , 1474720)** | **4818.4 (3896 , 5978.7)** | **2405797 (1696838 , 3263059)** | **5466.5 (3865.7 , 7367.7)** | **13.5 (-13.6 , 46.8)** |
| **Bahrain** | **24882 (19519 , 31869)** | **4911.1 (3964.2 , 6043.3)** | **93622 (66063 , 123860)** | **5720 (4016 , 7508.8)** | **16.5 (-9.2 , 49.1)** |
| **Egypt** | **2281368 (1796767 , 2855481)** | **4324.8 (3470.1 , 5364.4)** | **5481791 (3911372 , 7472414)** | **5213.9 (3730.1 , 7105.7)** | **20.6 (-6.9 , 52)** |
| **Iran** | **3523366 (3025159 , 4099970)** | **6760.7 (5887.3 , 7737.2)** | **7288282 (6217244 , 8378425)** | **8198.4 (7057.5 , 9426.2)** | **21.3 (14 , 28.7)** |
| **Iraq** | **846004 (663620 , 1067692)** | **4996.8 (4026.3 , 6145.9)** | **2460206 (1733541 , 3379014)** | **5837.9 (4136.6 , 7887)** | **16.8 (-10.7 , 48.3)** |
| **Jordan** | **173038 (135206 , 221038)** | **4902.5 (3986 , 6058.8)** | **737436 (513268 , 978697)** | **5694.7 (4016.1 , 7542)** | **16.2 (-10.3 , 47.6)** |
| **Kuwait** | **78853 (62012 , 100188)** | **4517.8 (3656.2 , 5571.1)** | **256068 (181744 , 346024)** | **5065.6 (3632.5 , 6817.4)** | **12.1 (-12.5 , 42.4)** |
| **Lebanon** | **176425 (140420 , 219239)** | **6081.1 (4914 , 7498.1)** | **473053 (331020 , 634186)** | **8274.7 (5766.6 , 11065.3)** | **36.1 (6.7 , 72.9)** |
| **Libya** | **202542 (159393 , 257900)** | **5035.8 (4089.8 , 6125.2)** | **458304 (328436 , 626545)** | **6188.3 (4448 , 8520)** | **22.9 (-2.6 , 53.8)** |
| **Morocco** | **1187842 (937985 , 1484508)** | **4844.5 (3919.7 , 5942.1)** | **2282348 (1643839 , 3088910)** | **5985.1 (4316.5 , 8093)** | **23.5 (-3 , 59.5)** |
| **Oman** | **85674 (67624 , 107669)** | **4662.3 (3772.1 , 5691.8)** | **271740 (189928 , 370977)** | **5624.3 (3947.3 , 7639.4)** | **20.6 (-7 , 50.9)** |
| **Palestine** | **96291 (75662 , 122697)** | **5295 (4256 , 6493.6)** | **327024 (230094 , 445128)** | **6302.6 (4552 , 8392.6)** | **19 (-9.1 , 53.8)** |
| **Qatar** | **20011 (15712 , 25556)** | **4435.2 (3583.9 , 5402.3)** | **155078 (107588 , 219879)** | **4892.2 (3499.4 , 6708.9)** | **10.3 (-15.2 , 41.1)** |
| **Saudi Arabia** | **695906 (540235 , 885948)** | **4584.7 (3698.5 , 5704.7)** | **2108231 (1495959 , 2885792)** | **5144.1 (3714.6 , 6929.1)** | **12.2 (-14 , 43.9)** |
| **Sudan** | **894634 (706554 , 1135003)** | **4860.7 (3938.6 , 6023.4)** | **2497205 (1789131 , 3544144)** | **5734.4 (4172.6 , 8075.9)** | **18 (-10.4 , 52.3)** |
| **Syrian Arab Republic** | **602714 (472650 , 766792)** | **5168 (4158.2 , 6416.8)** | **945251 (668283 , 1288268)** | **6427.4 (4549.3 , 8864.8)** | **24.4 (-5.6 , 59.1)** |
| **Tunisia** | **409818 (324256 , 513670)** | **5008.4 (4055.9 , 6110.6)** | **826676 (598155 , 1122049)** | **6875.4 (4987.4 , 9496.7)** | **37.3 (6.4 , 73.6)** |
| **Turkey** | **2316873 (2079163 , 2560140)** | **4059.7 (3683.8 , 4492.1)** | **4694266 (3283843 , 6411243)** | **5420.1 (3786.6 , 7370.4)** | **33.5 (-4.1 , 77.6)** |
| **United Arab Emirates** | **79359 (64650 , 98271)** | **4286.5 (3534.2 , 5169.8)** | **519114 (358345 , 711671)** | **5168.1 (3648.7 , 7062.6)** | **20.6 (-9.3 , 55.7)** |
| **Yemen** | **576267 (451780 , 736561)** | **4933.6 (3979.3 , 6133.3)** | **1702674 (1218061 , 2315536)** | **5203 (3785.3 , 6971.3)** | **5.5 (-18.3 , 34.3)** |

| **Table S3: Incidence of anxiety disorders in 1990 and 2021 and the percentage change in the age-standardised rates (ASRs) per 100,000 in the Middle East and North Africa region**  **(Generated from data available from http://ghdx.healthdata.org/gbd-results-tool)** | | | | | |
| --- | --- | --- | --- | --- | --- |
|  | **1990** | | **2021** | | **Percentage change in ASRs per 100,000** |
|  | **No (95% UI)** | **ASRs per 100,000 (95% UI)** | **No (95% UI)** | **ASRs per 100,000 (95% UI)** |  |
| **North Africa and Middle East** | **2583167 (2106821 , 3173584)** | **728.1 (606.9 , 902.1)** | **5702847 (4644052 , 7167442)** | **883.4 (722.4 , 1108.5)** | **21.3 (12.4 , 29.7)** |
| **Afghanistan** | **71863 (57817 , 90890)** | **722.3 (589.6 , 916.9)** | **303477 (212742 , 426472)** | **901.4 (640.4 , 1264.1)** | **24.8 (-2.7 , 54.7)** |
| **Algeria** | **189431 (152649 , 239456)** | **710.1 (583.1 , 897.2)** | **363903 (255478 , 506148)** | **812.8 (572.9 , 1135.8)** | **14.5 (-12.8 , 46.7)** |
| **Bahrain** | **3874 (3109 , 5030)** | **728.9 (592.2 , 916.1)** | **13861 (9613 , 18860)** | **884.6 (622.2 , 1191.8)** | **21.4 (-5.1 , 58.2)** |
| **Egypt** | **377619 (302045 , 471559)** | **660.5 (536.6 , 828.4)** | **899197 (636150 , 1243266)** | **813.5 (581.6 , 1118.2)** | **23.2 (-5.4 , 56.4)** |
| **Iran (Islamic Republic of)** | **558534 (459403 , 686612)** | **928 (765.8 , 1132.5)** | **986389 (808525 , 1216359)** | **1142 (938.6 , 1401.4)** | **23.1 (15.5 , 30.7)** |
| **Iraq** | **137998 (110290 , 175267)** | **718.3 (588.6 , 904.6)** | **373399 (263928 , 520561)** | **846.8 (608.5 , 1170.4)** | **17.9 (-8.5 , 50.3)** |
| **Jordan** | **28552 (22684 , 36609)** | **720.8 (586.5 , 912.6)** | **112466 (80016 , 156130)** | **852.5 (609.3 , 1182.4)** | **18.3 (-8.4 , 49.4)** |
| **Kuwait** | **12545 (10101 , 15952)** | **691.1 (564.5 , 865.6)** | **36403 (26141 , 50368)** | **761.4 (556.4 , 1056)** | **10.2 (-13.7 , 41)** |
| **Lebanon** | **25082 (20504 , 31627)** | **820.2 (670.8 , 1034.7)** | **63879 (45411 , 85516)** | **1168 (824 , 1570.7)** | **42.4 (10.8 , 80.1)** |
| **Libya** | **33052 (26353 , 41514)** | **735.9 (603.6 , 938.9)** | **65019 (45650 , 89924)** | **910.9 (645.2 , 1275.3)** | **23.8 (-2.4 , 54.4)** |
| **Morocco** | **188244 (151841 , 242204)** | **713 (581.7 , 905.2)** | **336701 (246909 , 452168)** | **894.6 (658.9 , 1198.9)** | **25.5 (-2.8 , 61.2)** |
| **Oman** | **14540 (11637 , 18443)** | **705.5 (570.7 , 892)** | **42559 (30713 , 57926)** | **869.9 (624.1 , 1158.1)** | **23.3 (-5.4 , 54.3)** |
| **Palestine** | **15790 (12501 , 19957)** | **750.6 (602.7 , 939.6)** | **51315 (36397 , 70569)** | **921.2 (657.7 , 1246.9)** | **22.7 (-6.9 , 58.7)** |
| **Qatar** | **3186 (2531 , 4075)** | **691.2 (569 , 863.7)** | **23539 (15859 , 32553)** | **773.4 (547.5 , 1069.2)** | **11.9 (-13.8 , 43.7)** |
| **Saudi Arabia** | **116423 (92020 , 146046)** | **696 (564 , 876.8)** | **310803 (223060 , 432305)** | **788.5 (572.3 , 1076.1)** | **13.3 (-12.8 , 44.6)** |
| **Sudan** | **147189 (116507 , 187214)** | **714 (581.6 , 910.9)** | **403701 (280070 , 577617)** | **858.7 (612.4 , 1211.3)** | **20.3 (-7.7 , 53.8)** |
| **Syrian Arab Republic** | **100132 (79516 , 126037)** | **746.6 (604.6 , 951.3)** | **134788 (96450 , 183583)** | **924 (659.9 , 1258.7)** | **23.8 (-4.6 , 57.7)** |
| **Tunisia** | **63850 (51192 , 81413)** | **728.5 (593.8 , 927.8)** | **118721 (83853 , 168901)** | **1025.7 (722.5 , 1475)** | **40.8 (7.9 , 80.6)** |
| **Turkey** | **379598 (319675 , 443584)** | **630.7 (530.4 , 738)** | **698666 (503758 , 964631)** | **841.3 (608.4 , 1151.4)** | **33.4 (-1.2 , 75.5)** |
| **United Arab Emirates** | **13218 (10592 , 16980)** | **675.6 (555.3 , 850.3)** | **79861 (54355 , 114045)** | **835.4 (606.5 , 1165.6)** | **23.7 (-6.4 , 60.2)** |
| **Yemen** | **101034 (80819 , 127491)** | **720.7 (584.6 , 914.4)** | **278881 (198872 , 387103)** | **769.1 (553.6 , 1044.7)** | **6.7 (-18.5 , 37.3)** |

| **Table S4: YLDs due to anxiety disorders in 1990 and 2021 and the percentage change in the age-standardised rates (ASRs) per 100,000 in the Middle East and North Africa region**  **(Generated from data available from http://ghdx.healthdata.org/gbd-results-tool)** | | | | | |
| --- | --- | --- | --- | --- | --- |
|  | **1990** | | **2021** | | **Percentage change in ASRs per 100,000** |
|  | **No (95% UI)** | **ASRs per 100,000 (95% UI)** | **No (95% UI)** | **ASRs per 100,000 (95% UI)** |  |
| **North Africa and Middle East** | **1908925 (1313103 , 2684061)** | **587.7 (407.2 , 806.3)** | **4508067 (2969187 , 6368321)** | **707.1 (469.8 , 994.9)** | **20.3 (11.8 , 28.3)** |
| **Afghanistan** | **52735 (35264 , 75431)** | **580.2 (391.3 , 805.4)** | **212963 (129925 , 320564)** | **708.3 (439.8 , 1058)** | **22.1 (-5 , 52.9)** |
| **Algeria** | **139381 (91894 , 198019)** | **574.4 (387.9 , 796)** | **286953 (176141 , 418472)** | **651 (400.8 , 949.5)** | **13.3 (-14.1 , 47.2)** |
| **Bahrain** | **3000 (1939 , 4311)** | **585.8 (387.8 , 814.5)** | **11201 (6731 , 16894)** | **682.8 (412.7 , 1045)** | **16.6 (-9.7 , 50.2)** |
| **Egypt** | **274447 (181692 , 393231)** | **515.3 (348.9 , 720.6)** | **658669 (380569 , 983989)** | **622.3 (363.7 , 915.9)** | **20.8 (-6.9 , 53.4)** |
| **Iran** | **423348 (292564 , 590245)** | **801.4 (555.5 , 1093.4)** | **864198 (587427 , 1191910)** | **973.7 (659.1 , 1345.8)** | **21.5 (14.1 , 28.7)** |
| **Iraq** | **101355 (66850 , 145982)** | **590.4 (396.7 , 818.6)** | **293838 (177147 , 444955)** | **690.9 (425 , 1028.3)** | **17 (-10.6 , 48.8)** |
| **Jordan** | **20939 (13811 , 30493)** | **584.2 (390.9 , 828.2)** | **88575 (52135 , 137122)** | **679.2 (404.1 , 1050.3)** | **16.3 (-10.6 , 47.5)** |
| **Kuwait** | **9535 (6203 , 13673)** | **541.4 (360.4 , 759.7)** | **30497 (18804 , 46382)** | **604.1 (377.5 , 926.5)** | **11.6 (-12.8 , 42.7)** |
| **Lebanon** | **21005 (13990 , 29732)** | **719.7 (483.4 , 1004.4)** | **55917 (33924 , 82475)** | **981 (592.8 , 1442.8)** | **36.3 (6.2 , 72.6)** |
| **Libya** | **24490 (15918 , 35259)** | **601.2 (399.9 , 845.7)** | **54530 (33340 , 80996)** | **735.5 (441.9 , 1099.2)** | **22.3 (-3.7 , 52.8)** |
| **Morocco** | **142403 (94744 , 203756)** | **575 (388.9 , 802.4)** | **270397 (168855 , 405942)** | **708.9 (442.7 , 1062.7)** | **23.3 (-3.5 , 58.2)** |
| **Oman** | **10362 (6699 , 14785)** | **556.3 (373.1 , 778.8)** | **32703 (19847 , 48698)** | **673.1 (412.2 , 1001.8)** | **21 (-7.1 , 52.1)** |
| **Palestine** | **11590 (7634 , 16754)** | **628.3 (422.9 , 887.3)** | **39245 (23650 , 60192)** | **747.8 (457 , 1112.1)** | **19 (-9.4 , 53.5)** |
| **Qatar** | **2415 (1571 , 3477)** | **530.4 (354.8 , 742.5)** | **18560 (11154 , 27035)** | **583.1 (363 , 829.7)** | **9.9 (-16.5 , 40.7)** |
| **Saudi Arabia** | **84002 (54257 , 121400)** | **545.5 (365.6 , 769.6)** | **251809 (157586 , 378772)** | **612.8 (381.2 , 918.2)** | **12.3 (-14.1 , 44.8)** |
| **Sudan** | **107315 (70577 , 154517)** | **576.2 (382.4 , 807)** | **300202 (185510 , 468843)** | **681.9 (428.1 , 1046.7)** | **18.3 (-10.8 , 53.6)** |
| **Syrian Arab Republic** | **72816 (47221 , 105802)** | **615.4 (414.7 , 863.5)** | **112090 (70647 , 167371)** | **759.8 (483.2 , 1145.4)** | **23.5 (-6.3 , 57)** |
| **Tunisia** | **49389 (32639 , 70157)** | **598 (398.5 , 840.4)** | **97771 (61489 , 144542)** | **816.8 (506.6 , 1206.5)** | **36.6 (5.6 , 73.7)** |
| **Turkey** | **279108 (197842 , 374262)** | **484.7 (343.1 , 650.7)** | **559351 (332345 , 830643)** | **648.1 (385.2 , 958.6)** | **33.7 (-3.8 , 77.5)** |
| **United Arab Emirates** | **9589 (6348 , 13644)** | **512.3 (350.3 , 712)** | **61832 (37158 , 96015)** | **618.4 (374.2 , 940.3)** | **20.7 (-9.5 , 58)** |
| **Yemen** | **68657 (44604 , 100042)** | **579.2 (386.9 , 805.7)** | **202561 (122312 , 306292)** | **611.7 (374.9 , 903.7)** | **5.6 (-18.4 , 34.1)** |

**
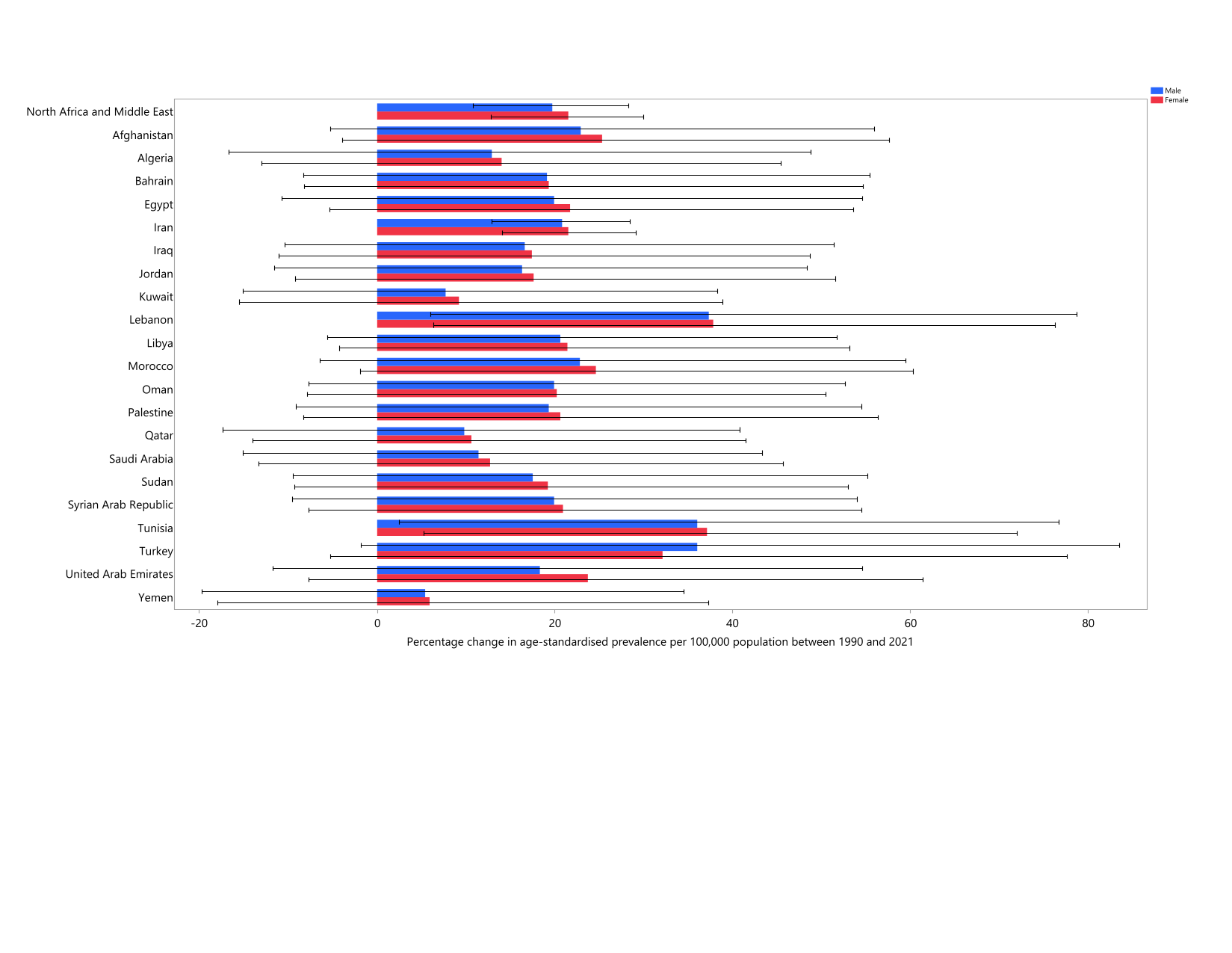
**

**Figure S1:** The percentage change in the age-standardised prevalence of anxiety disorders in the Middle East and North Africa region from 1990 to 2021, by sex and country. (Generated from data available from <http://ghdx.healthdata.org/gbd-results-tool>).


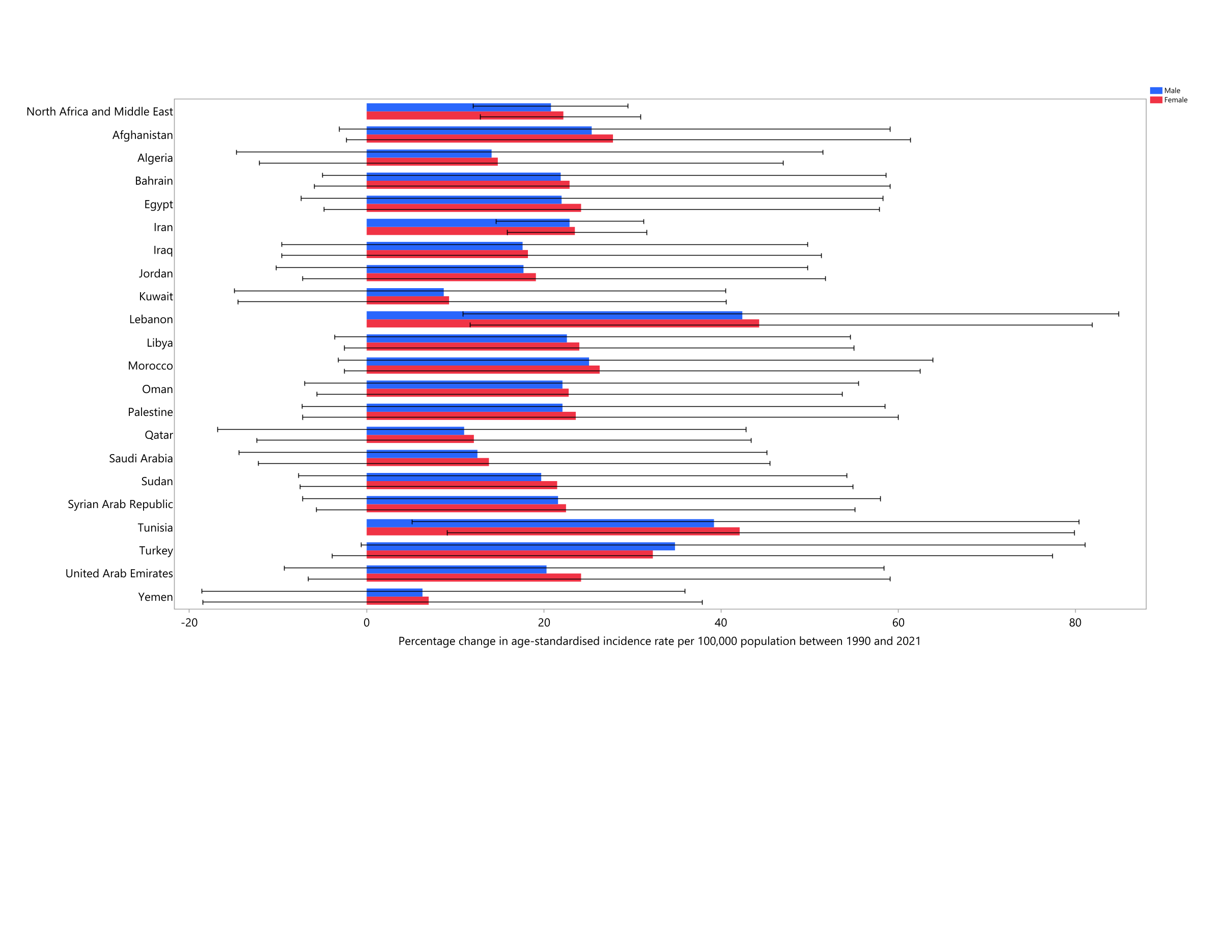


**Figure S2:** The percentage change in the age-standardised incidence rate of anxiety disorders in the Middle East and North Africa region from 1990 to 2021, by sex and country. (Generated from data available from <http://ghdx.healthdata.org/gbd-results-tool>).


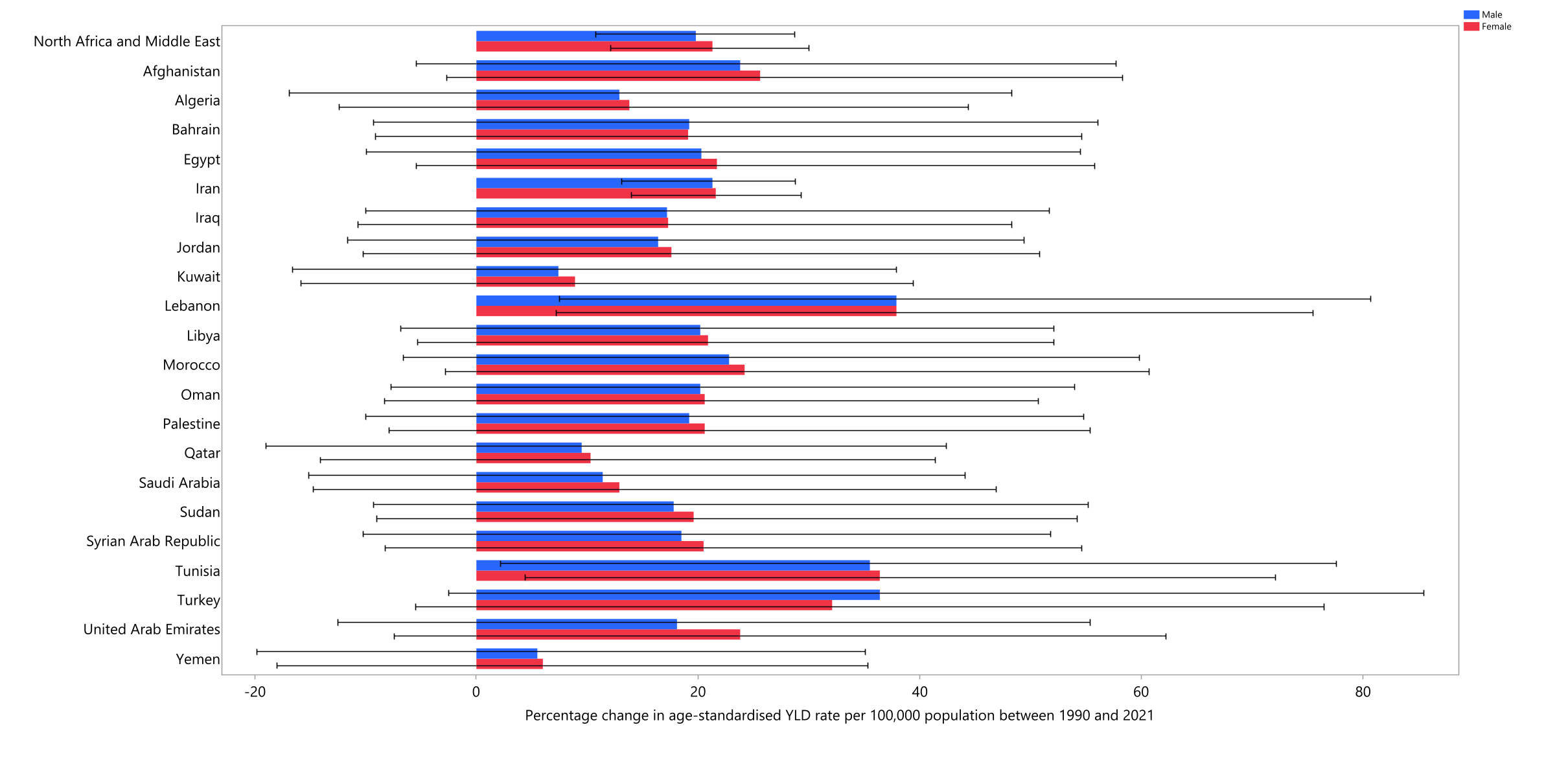


**Figure S3:** The percentage change in the age-standardised YLD rate of anxiety disorders in the Middle East and North Africa region from 1990 to 2021, by sex and country. YLD= years lived with disability (Generated from data available from <http://ghdx.healthdata.org/gbd-results-tool>).
